# Super-Aggregations of Krill and Humpback Whales in Wilhelmina Bay, Antarctic Peninsula

**DOI:** 10.1371/journal.pone.0019173

**Published:** 2011-04-27

**Authors:** Douglas P. Nowacek, Ari S. Friedlaender, Patrick N. Halpin, Elliott L. Hazen, David W. Johnston, Andrew J. Read, Boris Espinasse, Meng Zhou, Yiwu Zhu

**Affiliations:** 1 Nicholas School of the Environment and Pratt School of Engineering, Duke University Marine Laboratory, Beaufort, North Carolina, United States of America; 2 National Oceanic and Atmospheric Administration (NOAA) Pacific Fisheries Environmental Lab and University of Hawaii JIMAR, Pacific Grove, California, United States of America; 3 Laboratoire d'Océanographie Physique et Biogéochimique, Centre Océanologique de Marseille, CNRS, Université de la Méditerranée, Campus de Luminy, Marseille, France; 4 Department of Environment, Earth and Ocean Sciences, University of Massachusetts Boston, Boston, Massachusetts, United States of America; University of Glamorgan, United Kingdom

## Abstract

Ecological relationships of krill and whales have not been explored in the Western Antarctic Peninsula (WAP), and have only rarely been studied elsewhere in the Southern Ocean. In the austral autumn we observed an extremely high density (5.1 whales per km^2^) of humpback whales (*Megaptera novaeangliae*) feeding on a super-aggregation of Antarctic krill (*Euphausia superba*) in Wilhelmina Bay. The krill biomass was approximately 2 million tons, distributed over an area of 100 km^2^ at densities of up to 2000 individuals m^−3^; reports of such ‘super-aggregations’ of krill have been absent in the scientific literature for >20 years. Retentive circulation patterns in the Bay entrained phytoplankton and meso-zooplankton that were grazed by the krill. Tagged whales rested during daylight hours and fed intensively throughout the night as krill migrated toward the surface. We infer that the previously unstudied WAP embayments are important foraging areas for whales during autumn and, furthermore, that meso-scale variation in the distribution of whales and their prey are important features of this system. Recent decreases in the abundance of Antarctic krill around the WAP have been linked to reductions in sea ice, mediated by rapid climate change in this area. At the same time, baleen whale populations in the Southern Ocean, which feed primarily on krill, are recovering from past exploitation. Consideration of these features and the effects of climate change on krill dynamics are critical to managing both krill harvests and the recovery of baleen whales in the Southern Ocean.

## Introduction

Around Antarctica, the distribution of many predators has been linked to that of Antarctic krill (*Euphausia superba)* aggregations across a range of spatial and temporal scales. Super-aggregations of krill with densities greater than 1000 individuals/m^3^ and horizontal scales of 100s of meters to several kilometres have been reported in the past [1], [2]. However, there have been few reports of aggregations containing >100 individuals/m^3^ over the past 20 years [3]. Most of these swarms have been found in offshore waters in summer months and were often dominated juvenile krill [2]. Few studies have described the distribution and behaviour of krill in the coastal waters of the Antarctic Peninsula in autumn [3], [4], when adult krill are believed to migrate inshore to overwinter under the shelter of sea ice [5], [6].

A variety of predators, including penguins, seals, seabirds, and baleen whales rely heavily on Antarctic krill as a food resource. Several studies have described the ecological interactions between krill and penguins [7], [8] and krill and seals [9] in the waters around the Antarctic Peninsula during summer months and over spatial scales of hundreds of kilometers. Similarly, spatial relationships have been established between cetaceans and the overall abundance of krill in summer months [10]. Around the Antarctic Peninsula, Santora et al. [11] found fin and humpback whales were associated with different age classes of krill in the South Shetland Islands in summer months. During autumn, Friedlaender et al. [12], [13] quantified the meso-scale distribution pattern of whales in relation to krill biomass and patch structure in the continental shelf waters of the Antarctic Peninsula. All previous studies indicate a tight linkage between the distribution of baleen whales and krill in this region. To date, however, no research has examined linkages between the distribution of krill and whales at the onset of winter in Antarctica. Furthermore, only recently have researchers been able to consider both the ecological relationships among krill predators (e.g., niche overlap) and the potential effects of climate change on those relationships, specifically as they relate to the availability of their krill prey [14]. However, a greater understanding of the foraging ecology of baleen whales is required in order to thoroughly investigate these associations.

We undertook a multi-disciplinary study to elucidate the ecological relationships between krill and baleen whales relative to oceanographic processes in the embayments of the Antarctic Peninsula at the onset of winter. We were particularly interested in the nature of this relationship because of the hypothesized seasonal movement of krill to inshore waters around the Antarctic Peninsula in winter, rapid changes in the climate and sea ice patterns of the Antarctic peninsula [15], [16] and the known relationships between baleen whales and krill. During the course of this study we encountered a persistent super-aggregation of Antarctic krill and humpback whales, including the largest aggregation of krill reported in over 20 years and the highest density of humpback whales ever documented.

## Materials and Methods

All animal-related work reported in this manuscript was permitted under the U.S. Marine Mammal Protection Act by the National Marine Fisheries Service Permit 808–1735, the Antarctic Conservation Act Permit 2009-014, and Duke University Institutional Animal Care and Use Permit A041-09-02.

We measured water currents using a 153-kHz narrow-band Acoustic Doppler Current Profiler (ADCP; Teledyne-RDI) mounted on the ARSV *LM Gould*. To describe hydrographic conditions, chlorophyll-a concentration (an index of phytoplankton density) and density of small zooplankton, we used an integrated physical and biological sensor package: 2 pairs of conductivity-temperature-depth sensors (CTD, Seabird Electronics), a fluorometer (Wet Labs); a Laser In Situ Scattering and Transmissometer (Sequoia Scientific); and a Laser Optical Plankton Counter (Brooke Ocean Technologies). To quantify krill biomass and distribution we used: an integrated MOCNESS [17] for estimates of krill size and acoustic target strength; dual EK-60 fisheries echosounders (38 and 120 kHz, Kongsberg-Simrad) calibrated according to [18] and mounted on a zodiac for fine scale (10^0^ km) measurements of acoustic volume backscattering; and the ADCP echo intensity to define the meso-scale (10^1^ km) geographic extent of the krill distributions.

We calculated the total biomass of krill in the aggregations as the product of the mean density of krill estimated by the EK-60 from the fine scale survey and the area of krill aggregation estimated by the ADCP. We made EK-60 measurements throughout the area of the aggregations. For the EK-60 estimates, we used the inverse method to estimate biomass using 38 and 120 kHz echosounders with measured krill size and distorted-wave born approximation (DWBA) modeled target strength estimates [19]. To estimate the percentage of krill from acoustically detected schools, we subtracted the linear backscatter cross-section at 38 kHz from 120 kHz to identify krill [3], [20], [21].

To estimate whale density, we conducted visual line transect surveys along four north-south and four east-west tracklines (i.e., long shore and perpendicular) using standard line-transect methods [22] and analyzed sighting data with Distance (V5.0), using the conventional distance sampling analysis engine [23]. We measured three-dimensional whale diving behaviour by attaching a multi-sensor tag [24], [25], and recorded surface behaviours using standard behavioural sampling methodology [26]. To estimate the potential removal of krill by whales in the bay, we applied consumption rates from [27] to the estimated total number of whales sighted in our survey.

## Results

**Figure 1:**
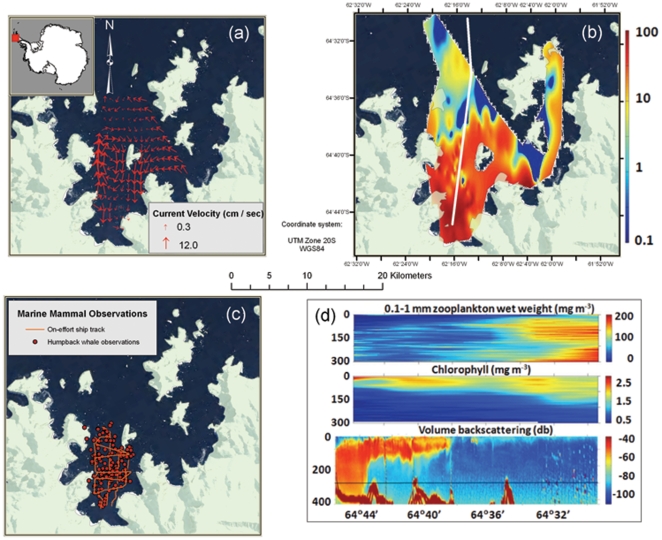
The physical and biological environment of Wilhelmina Bay, WAP, May 2009. Southerly katabatic winds and coastal currents produced the circulation field and retentive gyre shown in (a). Panel (b) shows the krill super-aggregation, scaled for biomass concentrations from 0.1–100 kg/m^2^. The white line indicates the transect followed to collect the data shown in (d). Humpback whale sightings (5.1 whales/km^2^) and surveys are shown in (c). Panel (d) shows meso-zooplankton (top), chlorophyll concentration (middle), and the vertical profile of the krill super-aggregation margin from south to north along the x-axis. High chlorophyll levels indicate a fall bloom; the lack of meso-zooplankton in the area of the krill aggregation is likely due to krill grazing.

On 1 May 2009 we entered Wilhelmina Bay, located along the WAP (Figure 1), to survey the distribution and abundance of krill and humpback whales. We encountered a large aggregation of krill and high densities of humpback whales. Over the next four weeks we documented the physical, chemical, and biological dynamics of this super-aggregation of krill and whales as well as the feeding ecology of the whales in relation to krill distribution and behaviour. This aggregation was not a singular event as we discovered smaller but still dense aggregations of krill and whales in Anvord Bay, located ca. 10 km south of Wilhelmina Bay.

A small cyclonic eddy occurred within Wilhelmina Bay driven by strong, episodic southerly katabatic winds (Figure 1a). The winds drove westward Ekman transport, which induced a geostrophic northward coastal current (20 cm/s) off the western coast of Wilhelmina Bay and upwelling at its southern and eastern margins. The eddy margins were nearly coincident with the spatial limits of the krill aggregation (Figure 1a,b). Throughout our study there was little sea ice; occasional brash ice covered <10% of the bay. We observed high chlorophyll-a levels (1.5 mg/m^3^) in the upper 100 m of the water column. Wet biomass of small zooplankton (in equivalent spherical diameters between 0.1 and 1 mm) varied from 150 mg/m^3^ outside the krill super-aggregation to 10 mg/m^3^ within it.

Individual krill sampled with the MOCNESS were 4.2±0.6 (SD) cm in length and contributed >99% of the biomass in net samples taken within the super-aggregation. The super-aggregation ranged from 10s of meters to 10s of km in the horizontal plane and from 8 to 410 m in the vertical, with a mean density of 130 g/m^3^ (170 individuals/m^3^) and a maximum density of 1500 g/m^3^ (2000 individuals/m^3^). From the fisheries acoustics (EK-60) data, we estimated an overall mean krill density of 62 g/m^3^. When this value was multiplied by the mean layer thickness and aggregation area estimated from the ADCP backscatter data, we estimated a total biomass of 2.0 million tons with krill comprising an estimated 88.4% of the total acoustic backscatter. This estimate represents the largest aggregation of krill reported in more than two decades even though significant effort has been invested in biological and physical sampling around the WAP over the last 20 years [28], [29], [30]. In Anvord Bay, we found a slightly smaller aggregation of an estimated 0.7 million tons of krill at similar densities.

**Figure 2:**
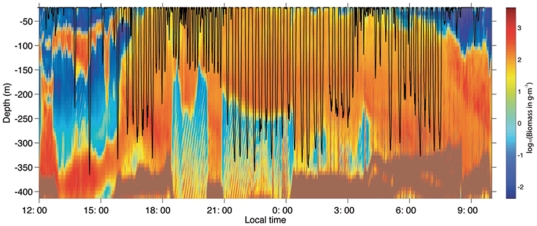
Humpback whale dive profile and krill biomass. Daytime resting behavior is indicated by the lack of dives. The exploratory deep dive at ∼1430 h local to 370 m is the deepest recorded dive for a humpback. The whale's diving behavior as measured by the DTag tracked the vertical movement of krill at night. Krill density was measured within 100 s of meters from the tagged whale (confirmed by surface observations and radio tracking).

In and around the Wilhelmina krill aggregation, we recorded 149 sightings of 306 humpback whales in 65 km of line transect surveys, with a density of 5.1 whales per km^2^ (%CV = 12.1). In Anvord Bay we estimated a density of 0.51 whales per km^2^. We tagged eleven whales in the two bays; all of these individuals rested and occasionally socialized during daylight and fed almost continuously throughout the night [25]. Whales typically began feeding by diving to depths of more than 300 m in the late afternoon, perhaps to sample the vertical distribution of krill (Figure 2). Tagged whales varied the depths of their feeding dives to: i) track diurnal changes in the vertical distribution of krill; ii) match changes in prey density at depth; and/or iii) forage along edges of the prey aggregation (Figure 2).

## Discussion

In the autumn adult krill migrate from offshore and continental shelf areas to inshore habitats where they remain through winter under the protective cover of sea ice [4]. Our findings support this hypothesized movement and suggest that krill may coalesce into very large super-aggregations in the autumn in the bays of the WAP [5]. The life history of Antarctic krill is intimately tied to sea ice [28]. Recruitment is related to the winter sea ice cover from the previous year, as diminished sea ice cover reduces habitat available for over-wintering juvenile and adult krill and reduces the size of the food-rich marginal sea ice zone in summer. Over the past 50 years, significant decreases in both total sea ice cover and the timing of winter sea ice advance around the WAP [15] have accompanied dramatic reductions in the standing biomass of krill [31], [32]. Interestingly, the Antarctic Peninsula supports extremely high krill biomass and predator densities in a region that experiences less sea ice than colder, adjacent regions of the Antarctic [6].

The density of humpback whales we observed was the highest point estimate ever reported, much greater than those derived from previous summer surveys in the adjacent Gerlache Strait [33], Western Antarctic Peninsula region [34], or the broader Antarctic [35]. Our estimates of whale density in Anvord Bay, an order of magnitude less than those we observed in Wilhelmina Bay, were similar to the highest densities reported in the past [33]. No previous systematic surveys of whales have been conducted in Wilhelmina Bay or in the northern WAP region during autumn.

Reilly et al. [27] calculated that a single humpback whale consumes 390–874 kg of krill per day. This estimate is the best available, though it likely represents a conservative approximation because it is based on basal metabolic rates and allometric relationships; furthermore, among other confounds, these whales in Wilhelmina are theoretically eating as much as possible to prepare for winter. Nonetheless, using Reilly et al. [27] we estimate that the 306 humpback whales we counted in Wilhelmina Bay consumed 60–134 metric tons per day, which represents a daily removal of only 0.003–0.007% of the biomass in the Wilhelmina Bay krill super-aggregation. Stammerjohn et al. [15] demonstrated that the current advance of winter sea ice around the Antarctic Peninsula is occurring 54 days later than in 1979. With this increase in the persistence of open water and thus the availability of prey, the whales we recorded in Wilhelmina Bay could potentially consume an additional 3,225–7,224 tons of krill (0.16–0.36% of the total biomass) during this period (Table 1).

**Table 1:** Estimated consumption of Antarctic krill by the 306 humpback whales we counted in Wilhelmina Bay, WAP during the 54-day change in the timing of sea ice advance described by Stammerjohn et al. [15] and using the daily consumption rate estimates from Reilly et al. [27].

daily consumption (kg)	total krill (kg)	tons consumed	model	Krill Abundance (tons)	% of total consumed by whales
390.34	6.45E+06	3225	Innes et al.	2.0E+06	0.161
497.23	8.22E+06	4108	Innes et al. revised	2.0E+06	0.205
694.38	1.15E+07	5737	2% max	2.0E+06	0.287
785.95	1.30E+07	6494	2.5% max	2.0E+06	0.325
874.33	1.44E+07	7224	3% max	2.0E+06	0.361

We used humpback whale abundance estimates from line transect surveys described in our results.

Changes in the physical structure of the marine ecosystem around the Antarctic Peninsula may have profound effects not only on the abundance of krill and baleen whales, but also on the ecological interactions among all krill predators and their prey. Our observations indicate that humpback whales and their prey co-occur in super-aggregations during late autumn in the bays and fjords along the WAP. Efforts to monitor the distribution, abundance and dynamics of these whales should account for these large aggregations. Furthermore, we must understand how changes in sea ice cover affect the feeding ecology of humpback whales and their competitors in the short-term and the dynamics of krill populations over the longer term, particularly given the increasing pressure from commercial krill harvests [36]. Failure to account for the effects of climate change on these dynamics will undermine our ability to understand changes in the standing biomass of Antarctic krill and also to predict the recovery of whale populations from a century of mismanagement and overexploitation [37].
